# Acetic and citric acids effect the type II secretion system and decrease the metabolic activities of salmon spoilage-related *Rahnella aquatilis* KM05

**DOI:** 10.1007/s11274-024-04101-z

**Published:** 2024-08-08

**Authors:** Kamila Myszka, Łukasz Wolko, Monika Borkowska

**Affiliations:** 1 Department of Biotechnology and Food Microbiology, Poznan University of Life Sciences, Wojska Polskiego 48, Poznan, PL-60-627 Poland; 2 Department of Biochemistry and Biotechnology, Poznan University of Life Sciences, Dojazd 11, Poznan, PL-60-632 Poland

**Keywords:** Spoilage of seafoods, *Rahnella aquatilis*, RNA-seq, Citric acid, Acetic acid, T2SS

## Abstract

*Rahnella aquatilis* causes seafoods to spoil by metabolizing sulfur-containing amino acids and/or proteins, producing H_2_S in products. The type II secretion system (T2SS) regulates the transport of proteases from the cytoplasm to the surrounding environment and promotes bacterial growth at low temperatures. To prevent premature fish spoilage, new solutions for inhibiting the T2SS of bacteria should be researched. In this study, global transcriptome sequencing was used to analyze the spoilage properties of *R. aquatilis* KM05. Two of the mapped genes/coding sequences (CDSs) were matched to the T2SS, namely, *qspF* and *gspE*, and four of the genes/CDSs, namely, *ftsH*,* rseP*,* ptrA* and *pepN*, were matched to metalloproteases or peptidases in *R. aquatilis* KM05. Subinhibitory concentrations of citric (18 µM) and acetic (41 µM) acids caused downregulation of T2SS-related genes (range from − 1.0 to -4.5) and genes involved in the proteolytic activities of bacteria (range from − 0.5 to -4.0). The proteolytic activities of *R. aquatilis* KM05 in vitro were reduced by an average of 40%. The in situ experiments showed the antimicrobial properties of citric and acetic acids against *R. aquatilis* KM05; the addition of an acidulant to salmon fillets limited microbial growth. Citric and acetic acids extend the shelf life of fish-based products and prevent food waste.

## Introduction

The global production of salmon-based products is constantly increasing worldwide because of their rich nutritional value and distinctive taste. In the European Union, nearly 60% of all salmon products are sold fresh (Rotariu et al. 2014). However, fresh salmon is a highly perishable product with a limited shelf life, which causes problems for suppliers, including economic losses related to outdated products and limited access to export markets (Aflaro et al. 2013).

To prevent seafood spoilage and to meet clean-label criteria, salmon fillets are vacuum-packed and stored at low temperatures; however, these conditions favor the growth of *Rahnella aquatilis* strains, and uncontrolled growth can contribute to adverse sensory changes and subsequent seafood spoilage. Studies by Alikunhi et al. (2014), Steenholdt Sørensen et al. (2020), and Myszka et al. (2023) showed that *R. aquatilis* is the dominant component of the microbiome of retail fish; bacteria were isolated from spoiled mullet, con, salmon and catfish fillets (Alikunhi et al. 2014; Steenholdt Sørensen et al. 2020; Myszka et al. 2023; Hickey et al. (2013). *Rahnella* spp. are involved in H_2_S production through the metabolism of sulfur-containing amino acids and/or proteins, which are widely believed to be the source of the fishy odor (Myszka et al. 2023; Yan et al. 2023; Remenant et al. 2015). The type II secretion system (T2SS) is directly involved in the transport of proteases from the cytoplasm to the surrounding environment; this system also promotes the growth of bacteria at low temperatures (Söderberg et al. 2004; Johnson et al. 2006). In the T2SS, proteins are first translocated across the inner membrane by the Sec or Tat pathway and then are transported from the periplasm to the exterior by an outer membrane secretin (Cianciotto et al. 2005). Although *R. aquatilis* has been confirmed to reduce the quality of fish, very few studies have attempted to characterize these new seafood hazards and their T2SS-related activities. A better understanding of the activity of *Rahnella* spp. on fish tissue is crucial for the overall economic and commercial viability of fish-based products. Mapping the molecular components of *Rahnella* spp. using high-throughput transcriptome sequencing (RNA-Seq) technology will facilitate the understanding of not only the role of the T2SS in the growth and/or microbial spoilage process of seafood but also the identification of key coding sequences (CDSs)/genes with altered expression that may lead to efficient inactivation of T2SS-related activities and the growth of bacteria in seafood. The food industry is forcing the search for alternative/clean label solutions to inhibit microbial growth/activities in food ecosystems.

Synthetic preservatives such as butylated hydroxyanisole (BHA), butylated hydroxytoluene (BHT) and *tert*-butylhydroquinone (TBHQ) possess wide antibacterial activity. However, since their use for fish preservation can cause certain health problems, replacing synthetic preservatives with alternative agents is of great concern (Salam 2007; Gokoglu 2018). There are many natural antimicrobial agents that are safe, easily obtainable, and of low commercial value and that can exhibit protective effects on seafood. Among them, organic acids deserve re-evaluation, especially for the treatment of new microbial food hazards. The acids present hydrophobic characteristics and consequently passively become soluble in the lipids of the cell membrane. Within the cytoplasm, a high pH facilitates the dissociation of the acid, which originates from ions that are unable to cross the membrane. This accumulation of ions causes simultaneous events that result in significant changes in metabolic activity and cell death (Baptista et al. 2020).

This study shed new light on the antimicrobial properties of citric acid and acetic acid and how they affect *R. aquatilis* in the context of a clean-label strategy and demonstrated the potential use of these agents in the preservation of salmon fillets. The following hypotheses were verified: (i) subMICs of citric and acetic acids influenced the T2SS, and (ii) subMICs of citric and acetic acids downregulated the expression levels of genes encoding proteases and thus decreased the proteolytic activities of *R. aquatilis* KM05. To describe the spoilage potential of *R. aquatilis* KM05, a global transcriptome of the strain was characterized, and the results indicated that the expression of all evaluated genes was related to the T2SS and the proteolytic activity of *R. aquatilis* KM05 under conditions simulating a food ecosystem.

## Materials and methods

### Strain and culture conditions

*R. aquatilis* KM05 was isolated from vacuum-packed salmon fillets purchased from a local market (Myszka et al. 2023). Sanger sequencing and PCR-RFLP analysis (restriction fragment length polymorphism) of the *16 S rRNA* were used for strain identification. *R. aquatilis* KM05 was deposited at the Culture Collection of the Department of Biotechnology and Food Microbiology, Poznan University of Life Sciences. The strain was preserved in cryovials (Medical Wire and Equipment, UK) and is freely available upon request.

The culture was carried out in modified Luria–Bertani (LB) media consisting of 0.5% (w/v) yeast extract (BD, USA), 1% (w/v) fish peptone (HiMedia, Thane, India), and 1% (w/v) NaCl (POCH, Poland) supplemented with subMICs of citric acid and acetic acid. The modified LB medium without addition of citric/acetic acid served as a control. The pH of the media was 5.0. The incubation process was carried out at 4 °C ± 2 °C for 5 days.

### Whole-transcriptomic analysis (RNA-seq) of *R. aquatilis* KM05

RNA-seq analysis was performed according to our previous work (Myszka et al. 2021). Briefly, an RNAqueous Kit (Thermo Fisher Scientific, USA) was used for total RNA isolation from *R. aquatilis* KM05 following the manufacturer’s recommendations. A Ribominus Transcriptome Isolation Kit (Invitrogen, USA) was used to remove excess ribosomal RNA. To construct the libraries, a Collibri™ Stranded RNA Library Prep Kit (Illumina, USA) and a Collibri™ H/M/R rRNA Depletion Kit (Invitrogen, USA) were used. The libraries were quantitatively and qualitatively assessed via a Qubit fluorimeter (Thermo Fisher Scientific, USA) and an electropherogram on a Bioanalyzer (Agilent, USA). Next-generation sequencing (NGS) was carried out on a MiSeq Illumina sequencer using a MiSeq Reagent Kit v3 (cycles) (Illumina, USA). The sequencing reads were processed using CLC Genomic Workbench v20 (Qiagen, USA). The reads were mapped to the corresponding genome assembly of *R. aquatilis* KM05 (NZ_CP034483) and normalized by the RPKM (reads per kilobase per million mapped reads). The RNA-seq data were deposited in the SRA NCBI data repository (Bioproject: PRJNA509367; Biosample: SRX9799400; SRA: SRR13376052).

### Determination of the antimicrobial activity of citric and acetic acids

The subMICs of citric and acetic acids toward *R. aquatilis* KM05 were evaluated via a broth microdilution method (CLSI 2012). Briefly, the tested organic acids were diluted in dimethyl sulfoxide (DMSO) (Sigma‒Aldrich, Merck KGaA, USA) to obtain a range of concentrations. Uninoculated modified LB medium without citric acid and acetic acid (Sigma‒Aldrich, Merck KGaA, USA) served as the control. Bacterial growth was measured during incubation at 4 °C. The first concentration that resulted in no significant growth inhibition (just below the MIC) was selected as the subMIC.

### Proteolytic estimation

To estimate the proteolytic activity of *R. aquatilis* KM05 following treatment with subMICs of citric and acetic acids, a method described by Polychroniadou (1988) was used. Briefly, 0.5 ml of the examined culture was added to 0.5 ml of buffer composed of 0.1 M Na_2_B_4_O_7_ in 0.1 M NaOH (POCH, Poland). Then, 1 ml (1 mg/ml) of 2,4,6-trinitrobenzenesulfonic acid (TNBS) (Sigma‒Aldrich, Merck KGaA, USA) was added. The samples were incubated at 37 °C for 1 h. Next, 2 ml of 0.1 M NaH_2_PO_4_ (POCH, Poland) containing 1.5 ml of Na_2_SO_3_ (POCH, USA) was added. The control probes were prepared with 0.5 ml of H_2_O. The absorbance at 420 nm on a SPECORD^®^ 205 UV‒VIS spectrophotometer (Analytic Jena AG, Germany) was measured. The percentage of proteolytic activity inhibition was calculated according to the following formula:

$$100 - (A/B\,\, \times \,100)$$

where A is the absorbance value obtained for *R. aquatilis* KM05 cultured on LB media supplemented with subMICs of acetic/citric acid and B is the absorbance value obtained for *R. aquatilis* KM05 cultured on LB media.

### RNA isolation and RT‒qPCR

*R. aquatilis* KM05 cultures were treated with RNAprotect Bacteria Reagent (Qiagen, USA). For total RNA isolation, a PureLink™ RNA Mini Kit (Ambion, USA) was used. The PureLink™ DNase Set (Ambion, USA) was used to remove residual DNA. RNA XR and IQ Assay Kits (Invitrogen, USA) were used for the quantification and quantification of RNA on a Qubit Fluorimeter 4 (Invitrogen, USA). Synthesis of the first strand of + cDNA was carried out via a High Capacity RNA-to-cDNA Kit (Life Technologies, USA). qPCR analysis was conducted in a CFX96 Touch Real-Time PCR Detection System (Bio-Rad, USA) with GoTaq^®^ Master Mix (Promega, Germany). Table 1 lists the primers used in the present study. The *16 S rRNA* was used as a reference gene. The cycle conditions were as follows: initial denaturation at 95 °C for 2 min and 45 cycles of denaturation at 95 °C for 15 s and annealing and extension at 58 °C for 1 min. The effect of subMICs of citric and acetic acids on the changes in the mRNA levels of genes encoding proteases and the T2SS in *R. aquatilis* KM05 were calculated by the 2^−ΔΔCt^ method (Schmittgen and Livak 2008).

**Table 1:** The oligonucleotides used in the RT-qPCR experiments

Primer	Sequence (5’→3’)	Amplified region
16S_F 16S_R	GGAGACTGCCGGTGACAAAC TGTAGCCCAGGCCGTAAGG	16 S rRNA gene
GSPF_F GSPF_R	AGCGGCGTTCCTTTACTTGA CAAAGAGTGAGCTGCCCTGA	*gspF* gene
GSPE_F GSPE_R	TACGAAAACTCTGCCCGCAT TACGAAAACTCTGCCCGCAT	*gspE* gene
FTSH_F FTSH_R	CGGCAAAAACGGTAACAGCA GCCGCTGAATTGCTCAAACA	*ftsH* gene
PTRA_F PTRA_R	GCTAGATGAGGAAGCCGACC AGTCAGCCTGGCAATAACCC	*ptrA* gene
RSEP_F RSEP_R	ATCTGTTCTGCAGGAGGTGC CAAACGACTGCCAGCTTTCC	*rseP* gene
PEPN_F PEPN_R	CCGCATTGTGGCGGATAAAG AACGGGTCCTGCCATTTGAT	*pepN* gene

### Antimicrobial assay in model salmon-based products

Fresh salmon fillets were purchased in a local supermarket (Poznan, Poland), washed in tap water (50^o^C) for 1 min, and cut into pieces using a sterile knife. The salmon pieces were dried with absorbent paper and then treated by UV-light (30 W, 50 cm irradiation distance) for 15 min for each side to eliminate any naturally present microbiota. Samples of raw salmon (10 g) were inoculated with 2 ml of *R. aquatilis* KM05 suspension containing 10^4^ CFU/ml. Fillets were subsequently marinated with olive oil containing subMICs of citric and acetic acids. Marinade supplemented with 0.001% benzoic acid served as a positive control. The marinades were poured over the salmon fillets. The samples were subsequently wrapped in polyvinyl chloride stretch films (Kraina Foils Packaging, Poland) and stored in an incubator (Thermo Fisher, USA) set at 4°C for 5 days.

The growth of *R. aquatilis* KM05 in model products was verified by the Koch plate method. Briefly, 10 g of model product was transferred to a sterile bag (Sigma‒Aldrich, Merck, KGaA, USA) filled with 90 ml of sterile saline and homogenized in a Pulsifier (Microgen Bioproducts, UK). Next, 1 ml of each serially diluted suspension was spread in duplicate on tryptic soy agar (TSA) Petri dishes (BD, USA). Petri dishes were incubated at 4 °C for 72 h. Salmon fillets treated with olive oil without the addition of examined acids served as a negative reference.

### Statistical analysis

The experiments were conducted in triplicate. The results are presented as the means ± standard deviations. Tukey’s parametric *post hoc* test was used to characterize the differences between datasets. *p* < 0.05 was considered to indicate statistical significance.

## Results

### RNA-seq data processing and analysis of differentially expressed CDSs/genes

The sequencing yield was 554.85 Mbp, and a cluster density of 97,000 per mm^2^ was achieved during sequencing. Q score values equal to 30 were obtained for 90.78% of the base reads, indicating good quality of the sequencing performed. The percentage of passes filtered (% PF) rate for the sequencing performed was 80.13%. Among the 98.36% of the mapped readings, 97.16% were paired. The distribution of distances between the ends of paired reads corresponding to the lengths of the sequenced RNA fragments ranged from 50 to 150 bp. In this work, the number of genes/CDSs to which the reads were mapped was 4376; 4373 genes/CDSs were differentially expressed in *R. aquatilis* KM05 incubated in modified LB medium. Using Pfam and Gene Ontology annotation, an analysis of the function of the transcripts was carried out; the Microbial Genomics CLC module was used for this purpose. The reference sequences were prepared by overlaying CDS annotations with the Find Prokaryotic Genes tool. Next, information about conserved domains, gene names and functions was assigned to the reading frames by selecting annotated CDSs with the Pfam Domains tool.

In this study, the transcribed CDSs/genes associated with metabolic processes involved in the T2SS and hydrolysis of T2SS-related proteins in cells were evaluated. A description of the selected CDSs of the *R. aquatilis* KM05 transcriptome is shown in Table 2. Two mapped genes/CDSs were matched to the T2SS: *qspF* and *gspE*. These genes/CDSs encode components of the T2SS required for the secretion of proteases. The FPKM values of *gspF* and *gspE* were 15.35 and 1.35, respectively. Four of the identified CDSs, namely, *ftsH*,* rseP*,* ptrA* and *pepN*, encode metalloproteases or peptidases in *R. aquatilis* KM05. The FPKM values of these CDSs were 79.9, 38.22, 10.05 and 17.43, respectively (Table 2).

**Table 2:** The description of selected CDS of the *Rahnella aquatilis* KM05 transcriptome. Reference genome no. NZ_CP034482

Name	Region	Fragment per kilobase million value	Gene length	Description
*gspF*	368008.369225	15.35	1218	component of T2SS
*gspE*	369222.370748	1.35	1527	component of T2SS
*ftsH*	507855.509798	79.9	1944	metalloprotease
*ptrA*	888075.890960	10.05	2886	endopeptidase
*rseP*	921241.922596	38.22	1356	metalloprotease
*pepN*	1737534.1740149	17.43	2616	aminopeptidase
*pmbA*	4442983.4444323	20.09	1341	metalloprotease

### subMIC

The antimicrobial effect of selected concentrations of acetic and citric acids against *R. aquatilis* KM05 was determined by measuring biomass growth. In this study, acetic acid (41 µM) and citric acid (18 µM) at sub-MIC concentrations were used for further experiments.

### Effects of subMICs of acetic and citric acids on the T2SS system of *R. aquatilis* KM05

To verify the anti-T2SS activity of acetic and citric acids at subMICs, RT‒qPCR experiments were carried out. In this work, the mRNA expression of genes encoding components of the T2SS was evaluated. *R. aquatilis* KM05 cultivated in modified LB media without organic acid supplementation served as a control. The transcriptional changes in the *gspF* and *gspE* were calculated as relative quantities normalized to the *16 S rRNA*.

In this study, the application of citric acid at subMICs to the culture medium decreased the expression of the *gspF* and *gspE* at all the cultivation time points, and the inhibition ranged from − 1.0 to -1.5 on average (Table 3). Similarly, acetic acid influenced the mRNA expression of all evaluated T2SS-related genes (see Table 3); the greatest reduction was observed on the 3rd and 5th days of cultivation, and the degrees of change were as follows for *gspF*: -2.0 and − 3.0, respectively, and for *gspE*: -3.0 and − 4.5, respectively. In this study, on the 1st day of incubation, the mRNA levels of *gspF* and *gspE* were − 1.0 each. In the *R. aquatilis* KM05 control samples, the expression of T2SS-related genes ranged from 0.4 to 1.1 (see Table 3).

**Table 3:** RT-qPCR confirmation of relative expression level ofT2SS-associated genes of *Rahnella aquatilis* KM05 grown in modified LB medium enriched with acetic and citric acids

Time of incubation (days)	LB medium supplemented with acetic acid		LB medium supplemented with citric acid		LB medium	
	Log_2_ (relative quantity)					
	gspF	gspE	gspF	gspE	gspF	gspE
1	-1.0	-1.0	-1.0	-1.0	0.4	0.8
2	-2.0	-3.0	-1.0	-1.0	0.9	1.1
5	-3.0	-4.5	-1.5	-1.0	0.9	1.1

### Effects of subMICs of acetic and citric acids on the proteolytic activity of *R. aquatilis* KM05

**Fig. 1:**
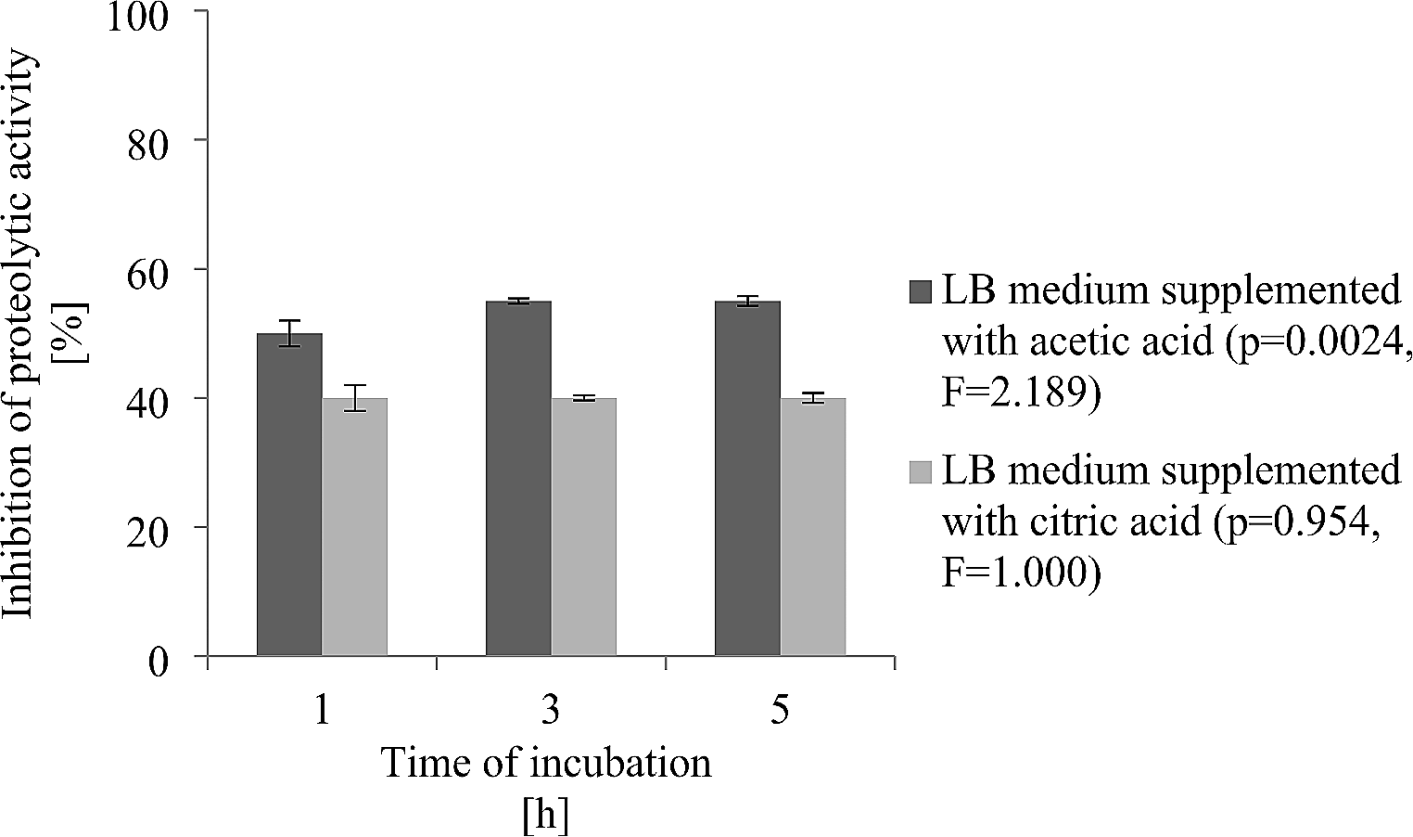
Inhibition of proteolytic activity of *Rahnella aquatilis* KM05 grown in modified LB medium with acetic and citric acids. The significance of the experimental data was determined by t-test (*p* < 0.05)

Spectrophotometric analysis with the TBNS reagent was carried out to identify changes in the proteolytic activity of *R. aquatilis* KM05 in vitro. Figure 1 shows the percentage inhibition of proteolysis after the cells were exposed to subMICs of acetic and citric acids. Organic acids impeded the proteolytic activity of *R. aquatilis* KM05 (*p* < 0.05); however, a greater reduction was detected in cells treated with acetic acid (approx. 55%). At subMICs, citric acid had a lower effect on the proteolytic activity of *R. aquatilis* KM05; proteolysis decreased to approximately 40% at all experimental time points (Fig. 1).

The results were then extended by verification of the mRNA levels of genes involved in the proteolysis of the bacteria. In this work, all studied genes were identified on the basis of RNA-seq data. The nondifferentially expressed reference *16 S rRNA* was used for estimation of the transcriptional levels of the *ftsH*,* ptrA*,* rseP*, and *pepN*. As presented in Table 4, all the genes were downregulated by the applied organic acids; however, the expression of these genes changed divergently. The greatest inhibition of the *ftsH* was observed after exposure to acetic acid. On the 3rd and 5th days of incubation of *R. aquatilis* KM05 on modified LB media enriched with acetic acid, the mRNA levels of the *ftsH* reached − 3.5 and − 4.0, respectively. Citric acid also downregulated all examined proteases; these changes ranged from − 0.5 to -1.0. In the *R. aquatilis* KM05 control samples, the mRNA levels of *ftsH*,* ptrA*,* rseP* and *pepN* ranged from 0.3 to 1.5 (Table 4).

**Table 4:** RT-qPCR confirmation of relative expression level of proteolytic activity-related genes of *Rahnella aquatilis* KM05 grown in modified LB medium enriched with acetic and citric acids

Time of incubation (days)	LB medium supplemented with acetic acid				LB medium supplemented with citric acid				LB medium			
	Log_2_ (relative quantity)											
	ftsH	ptrA	rseP	pepN	ftsH	ptrA	rseP	pepN	ftsH	ptrA	rseP	pepN
1	-1.5	-1.5	-1.5	-1.0	-0.5	-0.5	-0.5	-1.0	0.4	0.3	0.5	0.5
3	-3.5	-2.0	-2.0	-1.0	-0.5	-0.5	-1.0	-1.0	0.4	0.3	0.5	0.5
5	-4.0	-2.0	-2.5	-1.0	-1.0	-1.0	-1.0	-1.0	0.4	1.0	1.0	1.5

### Growth dynamics of *R. aquatilis* KM05 in model salmon-based products with subMICs of acetic and citric acids

In the first stage of the experiment, the salmon fillets were washed in tap water (50 ^o^C, 1 min) and sanitized by UV light. The mild heat pretreatment and UV light sterilization caused reduction of naturally existing psychrotrophic/psychrophilic bacteria on salmon fillets. No colonies (< 10 log CFU/g) were observed on TSA Petri dishes (data not shown).

The observed effect of the examined acids on the T2SS genes’ expression and T2SS-related proteolytic activity of *R. aquatilis* KM05 has forced continued research into the actual role of organic acids in fish preservation. In the present study, oil supplemented with acetic acid and citric acid at subMIC concentrations was used to verify the quality-enhancing properties of the tested fresh salmon fillets. The results were compared with those of the effect of benzoic acid on bacterial growth. The model salmon-based product was subsequently inoculated with standardized *R. aquatilis* KM05 culture and packed under vacuum conditions.

**Fig. 2:**
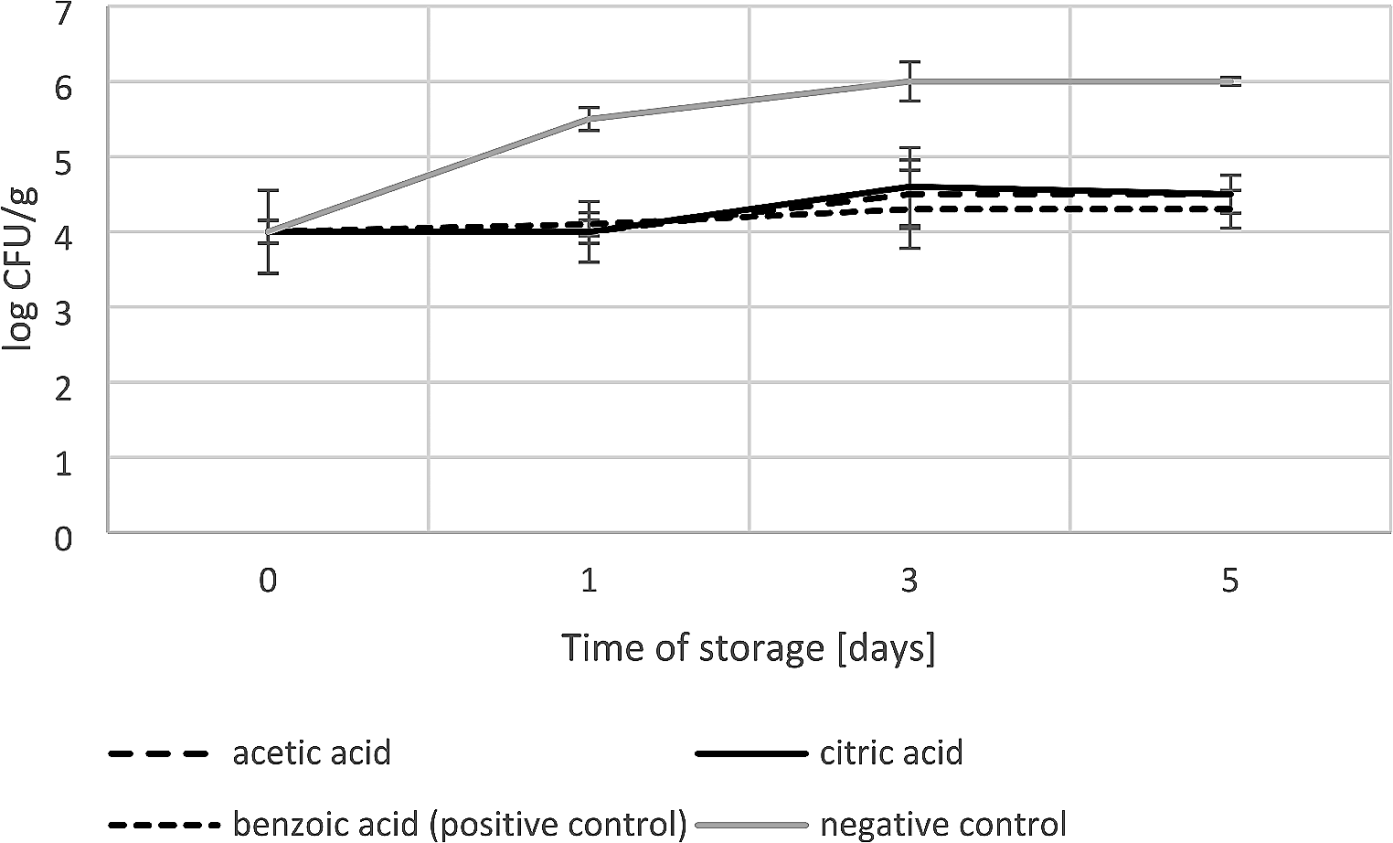
Effects of marinade and acetic/citric/benzoic acids on *Rahnella aquatilis* KM05 counts in salmon fillets stored at 4^o^C in vacuum conditions. Data points the mean values from three independent experiments. Error bars indicate the standard deviations (SD). The significance of the experimental data was determined by t-test (F = 45.34; *p* < 0.05)

The *R. aquatilis* KM05 biomass buildup during salmon fillets storage at 4 ^o^C is depicted in Fig. 2. After 1 day, the number of cells in the samples treated with subMICs of acetic acid and citric acid reached 4.0 log CFU/g and 4.1 log CFU/g, respectively. At the next point in the experiment (Day 3), the value increased to an average of 4.6 log CFU/g in both variants and, importantly, remained constant until the end of the experiment. A similar antibacterial effect was observed in samples treated with benzoic acid; after 1, 3 and 5 days of storage this agent impeded cell proliferation to 4.1 log CFU/g, 4.3 log CFU/g and 4.3 log CFU/g, respectively. The *R. aquatilis* KM05 control sample reached a critical spoilage value of 6 log CFU/g between 3 and 5 days (Fig. 2).

## Discussion

This study investigated the antimicrobial properties of citric acid and acetic acid. The use of these agents for preserving fresh fish fits perfectly with the clean label trend and the global drive to prevent premature food spoilage (Baptista et al. 2020). Efforts to control foodborne pathogens using organic acids have been reported; however, information regarding their effects on novel seafood threats, such as *R. aquatilis*, is lacking. Recent studies revealed that *Rahnella* spp. directly respond to the deterioration of retail cold-stored fish-based products (Steenholdt Sørensen et al. 2020; Myszka et al. 2023; Tavares et al. 2021). The frequency of isolation of *R. aquatilis* from commercially available foods indicates its resistance to commonly used synthetic fish preservatives.

The work started with RNA-seq analyses to clarify which metabolic pathway is activated during bacterial growth on a medium simulating a fish ecosystem and to select genes/CDSs that can play an important role in the spoilage of seafood. The downregulation of genes/CDSs encoding key cellular traits by food preservatives may ultimately improve the microbial status of products (Lamas et al. 2019). In this study, transcribed genes/CDSs were annotated to those encoding components of the T2SS (namely, *gspF* and *gspE*) and those involved in proteolysis (namely, *ftsH*,* ptrA*,* rseP*, and *pepN*) in *R. aquatilis* KM05. Among T2SS genes/CDSs, *gspE* encodes a cytoplasmic ATPase that provides the energy to power the T2SS, and *gspF* encodes a secretion channel (Green and Mecsas 2016). *ftsH*, *ptrA*,* rseP* and *pepN* encode proteases that can hydrolyze proteins and peptides for nutrition (Myszka et al. 2023; Wu and Chen 2011). Additionally, the RseP protease is involved in regulating intramembrane proteolysis (Kristensen et al. 2022). In that process, RseP cleaves a membrane-bound anti-sigma factor within the cell membrane, thereby mediating transmembrane signaling to trigger an adaptive response. In general, RseP is crucial for several biological processes, including stress response, sporulation, cell polarity, and virulence (Kristensen et al. 2022). Additionally, the FtsH protease is essential for the growth of bacteria, as was revealed for *Escherichia coli* strains (Akiyama et al. 1994); *ftsH* mutants of *Bacillus subtilis* were hypersensitive to heat and salt and defective in sporulation and division (Deuerrling et al. 1997).

The direct relationship between the T2SS and enzyme secretion is well described (Johnson et al. 2006; Cianciotto 2005), and mutations in genes encoding structural proteins of the T2SS affect the secretion of extracellular enzymes and bacterial growth (Sun et al. 2005). Moreover, downregulation of the *gspF* and *gspE* genes significantly changes the virulence of the *Enterobacter cloacae-rpoS* RNAi strain (Gao et al. 2022). Due to the vital role of T2SS-associated proteases and RseP and FtsH in the physiology of cells, these bacterial traits are regarded as attractive antimicrobial targets (Kristensen et al. 2022). Given the results of Lytou et al. (2019) showing that acetic and citric acids retard the growth of *Salmonella* in chicken fillets, it was hypothesized that these organic acids could affect the T2SS of *R. aquatilis* and could extend the shelf life of fish products. The undissociated part of weak organic acids penetrates bacterial cell membranes, and their intracellular dissociation reduces the cytoplasmic pH, affecting the metabolic activity of the cell (Lytou et al. 2019).

In the work, 41 µM and 18 µM acetic acid and citric acid, respectively, exhibited antimicrobial activity against *R. aquatilis* KM05. This is the first work evaluating the activity of these agents against the fish spoiler *Rahnella* spp. To date, acetic acid and citric acid MIC values have mainly been determined for foodborne *Shigiella* spp., *E. coli* and *Staphylococcus aureus* (Ye-Won et al. 2013; de Castro et al. 2023). In the study of Ye-Won et al. (2013), the MICs of acetic and citric acids against *Shigiella* spp. were 200 ppm and 300 ppm, respectively. According to Ye-Won et al. (2013), acetic acid produced greater proportions of injured cells than citric acid. The work of de Castro et al. (2023) showed that the MICs of acetic acid and citric acid against *E. coli* and *S. aureus* were 4.8 µM and 124.8 µM, respectively. MIC values vary depending on the specific strain and culture conditions (Mouton et al. 2018); however, this work provides further evidence that acetic acid and citric acid can preserve seafood and limit the growth of *Rahnella* spp.

Next, we verified the effect of sublethal acetic acid and citric acid concentrations on the mRNA levels of genes encoding components of the T2SS in *R. aquatilis* KM05. All of the examined agents inhibited the expression of T2SS genes in the *R. aquatilis* KM05 strain; however, the greatest reduction was observed for subMICs of acetic acid on the 3rd and 5th days of cultivation, and the degrees of change were as follows: *gspF*: -2.0 and − 3.0, respectively; and *gspE*: -3.0 and − 4.5, respectively. These results are in line with the observation of Lin et al. (2024), who also determined the anti-T2SS properties of phenyllactic acid. The MIC of phenyllactic acid downregulated *epsDEFGIJKLM* by 1.04–1.86 log2 (FC; fold change) in *Vibrio parahaemolyticus* ATCC17802 (Lin et al. 2024). In that strain, the *epsG*,* epsH*,* epsI*,* epsJ* and *epsK* encode T2SS of *Pseudomonas* spp.; *epsD* encodes a core component of the outer membrane complex, which controls the closure and opening of the outer membrane protein during secretion. The inner membrane platform of the T2SS is composed of three proteins encoded by *epsL*,* epsM* and *epsF* (Lin et al. 2024). The proteins secreted by the T2SS may include proteases, lipases, pectinases and toxins (Lin et al. 2024; Tomaś et al. 2022); in *V. parahaemolyticus* ATCC17802, the secretion of thermostable hemolysins mainly depends on the T2SS, and the disruption of the system by phenyllactic acid may reduce bacterial virulence (Lin et al. 2024).

Microbial enzymes are major causes of quality deterioration and food spoilage; thus, controlling the enzymatic processes that take place in seafood is required for extending the shelf life of the products (Xie et al. 2018). Bacterial proteolytic activity increases the accumulation of volatile aromatic compounds such as mercaptans, H_2_S or volatile acids in the product (Xie et al. 2018). The results of our earlier work, in which we demonstrated the effect of phenolic acids on the proteolytic activity of *Rahnella* spp., were the impetus for the search for yet other solutions that effectively inhibit the metabolic activity of these microorganisms (Myszka et al. 2023). This work revealed that *R. aquatilis* KM05 produced proteolytic enzymes at 4 °C. However, with low levels of mRNA genes encoding the components of the T2SS, a significant reduction in protease synthesis and downregulation of the *ftsH*,* ptrA*,* rseP*, and *pepN* were observed. These observations are in line with the work of Lelis et al. (2019), wherein both *gspD* and *gspE* mutants exhibited substantially less proteolytic activity than did the wild-type *Brukholderia glumae* (*gspD* and *gspE* encode an outer membrane secretin and a cytoplasmic ATPase of the T2SS, respectively, in bacteria). Additionally, the Δ*gspD* strain of *Acinetobacter* spp., which carries a deletion in the *gspD* inactivating the T2SS, does not secrete any T2SS substrates (Harding et al. 2016).

Finally, the antimicrobial properties of citric and acetic acids against *R. aquatilis* KM05 in seafood ecosystems were verified. In this study, via the addition of an acidulant to salmon fillets, we evaluated microbial growth limitations. Compared with the negative controls, the marinates containing subMICs of citric and acetic acids significantly delayed *R. aquatilis* KM05 growth beginning on the 1st day of storage of the model product. In those experiments, the number of cells in the samples treated with subMICs of the agents did not exceed 4.6 log CFU/g. In the negative control samples, *R. aquatilis* KM05 reached the critical spoilage value of 6 log CFU/g between 3 and 5 days of storage. The similar antimicrobial effect was noticed in samples treated with the synthetic preservative benzoic acid. In this work, the agent impeded cell proliferation to 4.1 log CFU/g − 4.3 log CFU/g. The antimicrobial effects of citric acid on *S. aureus* ATCC25923 and *E. coli* ATCC25922 were also confirmed by Li et al. (2023). Moreover, citric acid inhibited biofilm formation by *E. coli* and cellular activity within the *E. coli* biofilm, further highlighting the potential for broad application of organic acids in food technology (Ji et al. 2023).

In conclusion, the present work provides updated information related to the antimicrobial activity of citric and acetic acids. The use of the above agents in fish preservation is in line with the current trends of clean labels and the use of natural preservatives in food processing. Citric and acetic acids effectively inhibit the growth and metabolic activity of *R. aquatilis*, which poses a new threat to food quality and safety. The use of these compounds at subMIC concentrations extends the shelf life of fish-based products and prevents food waste. It is advisable to continue research on the practical use of organic acids in food technology.

## Data Availability

The RNA-seq data were deposited in the SRA NCBI data repository (Bioproject: PRJNA509367; Biosample: SRX9799400; SRA: SRR13376052) are available at the following URL: https://www.ncbi.nlm.nih.gov/sra.
